# Engineering the *Bacillus* Transcription Factor CcpC to Construct Citrate-Responsive Biosensors in *Escherichia coli*

**DOI:** 10.1021/acssynbio.6c00278

**Published:** 2026-05-26

**Authors:** Xinyu Gong, Shuo Yu, Jiyang Zhang, Zebang Chen, Jianli Zhang, Qi Gan, Yajun Yan

**Affiliations:** School of Chemical, Materials and Biomedical Engineering, College of Engineering, The University of Georgia, Athens, Georgia 30602, United States

**Keywords:** catabolite control protein C (CcpC), citrate, transcription factor (TF), hybrid promoter, protein engineering, biosensor

## Abstract

The tricarboxylic acid (TCA) cycle is an essential part of the central metabolic hub that provides energy and biosynthetic precursors. Efficient regulation of central carbon flux is critical for maintaining optimal productivity of microbial cell factories (MCFs). However, biosensors capable of sensing TCA intermediates remain limited. Here, we engineered the catabolite control protein C (CcpC) from *Bacillus* species to reconstruct citrate-responsive biosensors in*Escherichia coli*. Through hybrid promoter engineering, we systematically characterized and identified the functional roles of two CcpC binding sites. By applying the hybrid promoter, the engineered biosensor BcCcpC-PLBs exhibited the broadest dynamic range and highest expression level among its counterparts. Ligand profiling revealed the diverse responsiveness of BcCcpC to multiple metabolites of the TCA cycle. By structure-guided mutagenesis of BcCcpC, the obtained variant BcCcpC­(S138L) exhibited an improved dynamic range of up to 3.02-fold under 80 mM citrate induction. This work establishes the first transcription factor (TF)-based citrate-responsive biosensor, which broadens the regulatory toolkit for central metabolism engineering.

## Graphical abstract

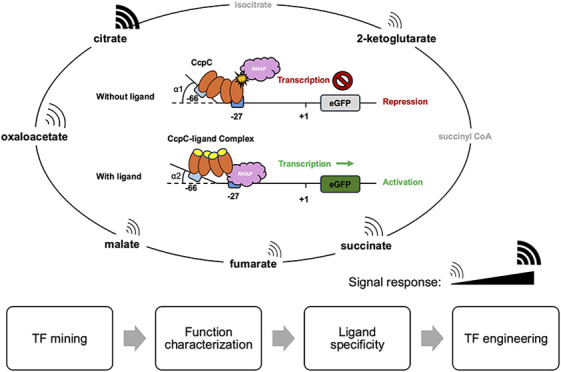

## 1 Introduction

Microbial cell factories (MCFs) serve as programmable platforms for the sustainable production of value-added compounds.^1,2^ Achieving high productivity often requires system-level metabolic rewiring to balance precursor supply, energy generation, and pathway flux.^3,4^ However, carbon competition between central metabolism and heterologous production pathways frequently limits metabolic efficiency.^5^ Developing robust regulatory tools that dynamically sense and respond to intracellular metabolic states remains a key challenge in optimizing MCFs performance. Metabolite-responsive biosensors have emerged as powerful tools for pathway dynamic regulation, high-throughput screening, and directed evolution without external intervention.^6–9^ By coupling gene expression to intracellular metabolite levels, biosensor-aided systems enable precise and programmable control over metabolic flux. While several transcription factor (TF)-based biosensors targeting central metabolites have been developed,^10–14^ biosensors capable of sensing citrate are unexplored.

Central carbon metabolism consists of interconnected pathways, including glycolysis, the pentose phosphate pathway, and the TCA cycle. The TCA cycle serves as a critical part of central carbon metabolism, providing energy and biosynthetic precursors for cellular metabolism.^15^ Citrate, the first intermediate of the TCA cycle, functions as a key regulatory node in both mammalian and microbial systems.^16–19^ In mammalian cells, citrate links mitochondrial metabolism to cytosolic biosynthesis and epigenetic regulation. To monitor citrate dynamics, genetically encoded biosensors based on the citrate-responsive histidine kinase CitA have been developed into FRET-based, intensiometric, and fluorescence lifetime-based formats,^20–23^ leveraging ligand-induced conformational changes for fluorescence readouts. In microbial systems, intracellular citrate levels are closely associated with glycolytic activity, pentose phosphate pathway flux, and overall TCA cycle dynamics, thereby influencing carbon distribution and product yields.^17,18,24^ Current metabolic engineering strategies typically rely on static manipulation of citrate synthase expression to modulate citrate levels.^25,26^ However, genetic biosensors capable of sensing intracellular citrate remain scarce in microbial systems, limiting the development of dynamic regulatory strategies targeting the TCA cycle.

Catabolite control protein C (CcpC) is a LysR-type transcriptional regulator (LTTR) widely distributed in Gram-positive bacteria and functions as a key regulator of TCA branch enzymes.^27^ In *Bacillus*, CcpC controls the transcription of citZ (citrate synthase), citB (aconitase), and citC (isocitrate dehydrogenase) in response to intracellular citrate levels.^28^ Mechanistically, CcpC binds to two binding sites within the *PcitB* promoter region, including a dyad symmetry element centered around −66 and a second half-dyad element near −27 relative to the transcription start site.^29^ Binding of CcpC dimers triggers DNA bending and blocks RNA polymerase (RNAP) access to the promoter, thereby repressing gene expression under low citrate conditions (Figure 1a). Upon citrate accumulation, ligand binding reduces the affinity of CcpC with one of the binding sites, relaxing the DNA bending and allowing transcriptional activation. Structurally, CcpC belongs to the canonical LTTR family, consisting of an N-terminal DNA-binding domain and a C-terminal inducer-binding domain. Crystal structure analysis of CcpC from *Bacillus amyloliquefaciens* (PDB: 7DMW) indicates that citrate binds within the C-terminal domain and is stabilized by multiple hydrogen bonds, which likely trigger conformational changes that regulate DNA binding and transcriptional control.^30^

**Figure 1:**
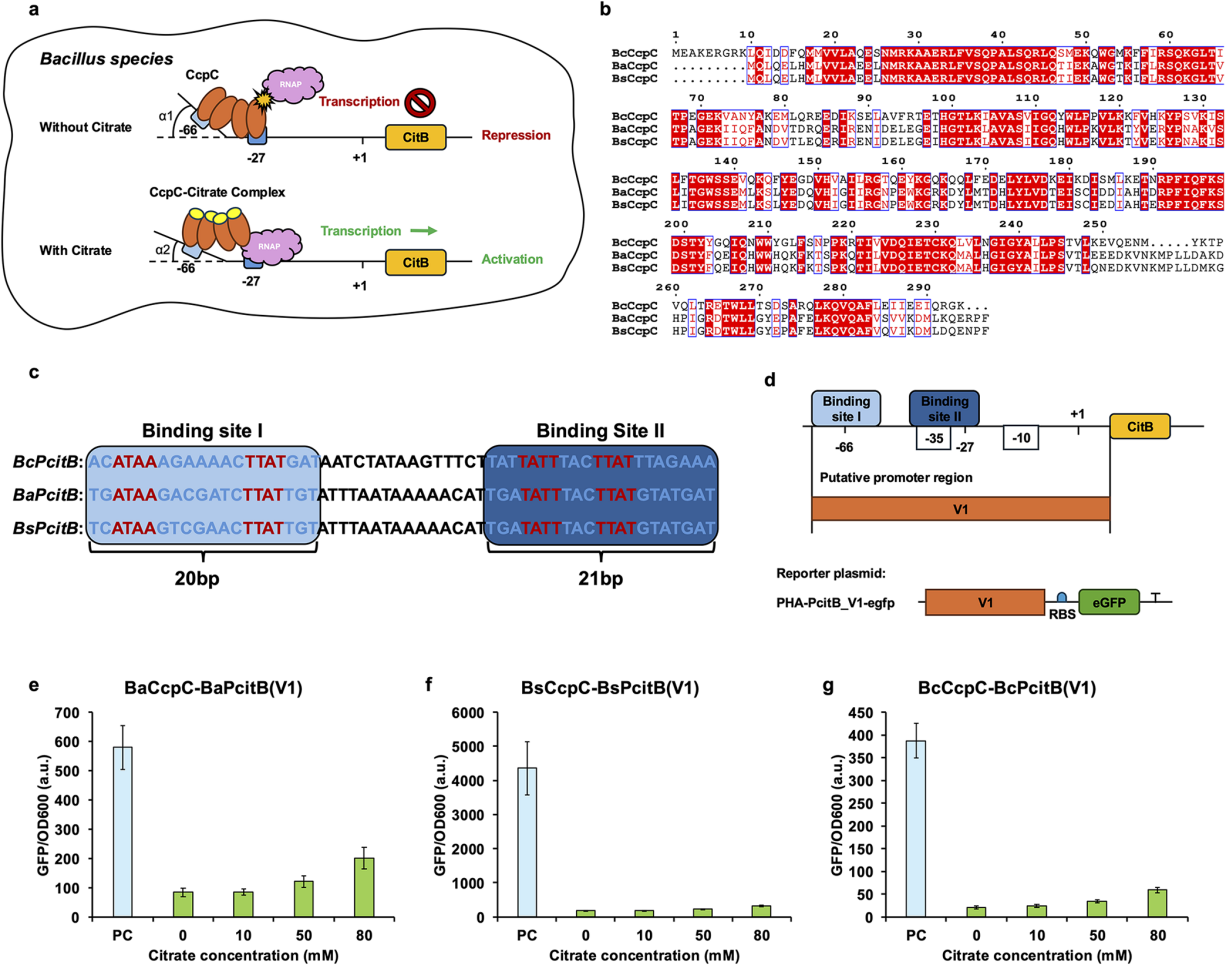
Mining and reconstruction of CcpC-based biosensor systems. (a) Proposed regulatory mechanism of the CcpC transcription factor in *Bacillus* species. In the absence of citrate, CcpC binds to two binding sites in the *PcitB* promoter located near positions −27 and −66, inducing DNA bending (α1) and blocking RNA polymerase (RNAP) recruitment, thereby repressing transcription. Upon citrate binding, the CcpC-citrate complex partially dissociates from the −27 binding site, reducing DNA bending (α2<α1) and allowing RNAP recruitment, which activates transcription. (b) Multiple sequence alignment of CcpC homologues from three *Bacillus* species. (c) Sequence alignment of the two conserved CcpC binding sites (binding site I&II) within the *PcitB* promoter from three *Bacillus* species. (d) Architecture of the *PcitB* promoter and design of the GFP reporter plasmid used for initial biosensor characterization. (e–g) Characterization of CcpC-*PcitB* biosensor systems derived from three *Bacillus* species. Relative GFP fluorescence (GFP/OD600) was measured under different citrate concentrations (0, 10, 50, and 80 mM). PC indicates the positive control strain in which the reporter is expressed without CcpC repression. Data represent mean ± SD from three biological replicates (*n* = 3).

*Escherichia coli*­(*E. coli*) is a well-established chassis for synthetic biology tool development due to its genetic tractability, rapid growth, and well-characterized metabolic network. In this study, we engineered CcpC homologues from three *Bacillus* species to construct citrate-responsive TF-based biosensors in*E. coli*. Initial reconstruction using the native *PcitB* promoters enabled citrate-responsive activation but resulted in limited fluorescence output. To address this limitation, we systematically characterized the functional roles of CcpC binding sites through hybrid promoter engineering. Our results demonstrated that binding site I functions as a stable anchoring site for CcpC, whereas binding site II acts as a citrate-responsive regulatory switch that modulates transcription via citrate-induced dissociation. Based on these mechanistic insights, we optimized the biosensor systems and identified BcCcpC-PLBs as the most effective variant. The engineered biosensor exhibited responsiveness to multiple TCA cycle metabolites and was further improved through structure-guided protein engineering of the BcCcpC regulator. Together, this work first establishes citrate-responsive CcpC-based biosensors in*E. coli* and provides a foundation for engineering metabolite-responsive regulatory systems for central metabolism.

## 2 Results

### 2.1 Mining and Identification of Citrate-Responsive TFs from *Bacillus*

CcpC is a LysR-type TF from *Bacillus* species that regulates genes involved in the TCA cycle, including aconitase (*citB* or *acnA*). In the absence of citrate, CcpC dimers bind to the operators of *PcitB* promoter and block RNAP access to the −35 region, resulting in transcription repression. In response to citrate, ligand binding to CcpC relieves the repression and enables RNAP access, thereby activating gene expression^28,29^ (Figure 1a). This citrate-responsive regulatory behavior suggests that CcpC is a promising candidate for developing citrate-responsive biosensors.

To explore citrate-responsive regulatory modules, we performed cross-species genome mining to identify CcpC homologues from different *Bacillus* species. Three representative homologues, including BaCcpC (WP_013352078.1), BsCcpC (BSU14140, BioCyc), and BcCcpC (WP_002026259.1), were selected from *B. amyloliquefaciens* *ATCC 23350*, *Bacillus subtilis* *168*, and *Bacillus cereus* *ATCC 14579*, respectively. Notably, the regulatory role of BcCcpC had not been previously investigated. For each homologue, the corresponding operators were identified from the upstream regions of *citB* or *acnA* promoters. Protein sequence alignment revealed that BaCcpC and BsCcpC share approximately 95% sequence identity, suggesting highly conserved regulatory functions, whereas BaCcpC and BcCcpC share approximately 53% sequence identity, indicating more distant homology while likely retaining similar regulatory roles (Figure 1b). To further investigate operator features, the upstream *PcitB* promoter regions were analyzed to identify putative CcpC binding sites. Two conserved operator motifs (binding site I&II) were identified, exhibiting interrupted dyad symmetry sequences ATAA­(N7)­TTAT and TATT­(N3)­TTAT, respectively (Figure 1c). The conservation of these motifs across different *Bacillus* species suggests a shared regulatory module that might be exploited for constructing citrate-responsive biosensors.

### 2.2 Comparative Reconstruction of Citrate-Responsive Regulatory Modules in *E. coli*

Based on the identified CcpC homologues and their corresponding *PcitB* promoters, we sought to comparatively characterize their citrate-responsive regulatory effects. To this end, the regulatory systems were reconstructed in the model chassis *E. coli*. The coding sequences of *Ba*CcpC, *Bs*CcpC, and *Bc*CcpC, together with their corresponding promoters *BaPcitB*, *BsPcitB*, and *BcPcitB*, were amplified from the genomes of *B. amyloliquefaciens*, *B. subtilis*, and *B. cereus*, respectively. The CcpC regulators were placed under the control of the IPTG-inducible promoter *pLlacO1* in the medium-copy number plasmids pMK-MCS to generate regulatory plasmids. For the reporter plasmids, promoter regions (V1) spanning from binding site I to the start codon of the *citB* or *acnA* gene were placed upstream of eGFP in high-copy plasmids pHA-eGFP-MCS (Figure 1d). This two-plasmid system constituted the initial CcpC biosensors and allowed independent tuning of genetic elements.

To assess citrate responsiveness, the regulatory and reporter plasmids were cotransformed into *E. coli* BW25113. CcpC expression was induced with 0.5 mM IPTG, and citrate was supplemented into the culture medium at gradient concentrations (0, 10, 50, and 80 mM). A reporter-only strain lacking the CcpC expression cassette was constructed to evaluate basal promoter activity (denoted as PC). Fluorescence assays revealed that the reconstructed biosensor systems exhibited citrate-dependent activation (Figure 1e–g). Importantly, comparative characterization of the three homologous CcpC-*PcitB* pairs indicated similar regulatory behaviors. The BaCcpC-BaPcitB and BcCcpC-BcPcitB systems showed relatively low basal fluorescence but exhibited clear citrate-dependent activation with 3.29- and 2.87-fold dynamic ranges upon 80 mM citrate induction, respectively (Figure 1e,g). In contrast, the BsCcpC-BsPcitB system exhibited higher basal fluorescence, but only a modest 1.77-fold dynamic range upon 80 mM citrate induction. (Figure 1f). These results demonstrated that although the CcpC regulatory mechanism is conserved across *Bacillus* species and functional in *E. coli*, the reconstructed biosensor systems exhibited distinct response strengths. These observations in basal expression and dynamic range motivated further engineering of the heterologous promoters to improve biosensor performance.

### 2.3 Characterization of CcpC Binding Sites through Hybrid Promoters

Previous studies have shown that CcpC binds to two binding sites within the *PcitB* promoter region through recognizing a dyad symmetry element centered around −66 and a second half-dyad element near −27 relative to the transcription start site.^29^ The binding of CcpC dimers to these sites results in DNA bending, which blocks RNAP access and leads to transcriptional repression (Figure 1a). Upon citrate induction, the DNA bending angle is reduced as citrate binding alters the regulator-operator interaction. In this state, the CcpC-citrate complex dissociates from binding site II while remaining associated with binding site I. This conformational change exposes the −35 region and activates transcription initiation by improving RNAP accessibility and facilitating its recruitment. In addition, the interaction between CcpC complex and RNAP may further enhance RNAP recruitment. To experimentally validate the contribution of these key genetic elements, we extracted them from their native promoter context and implemented them into constitutive promoters in a plug-and-play manner for functional validation. Accordingly, a series of hybrid promoters containing different combinations of two binding sites was designed and functionally characterized through the hypothesis-driven method.

To investigate whether the CcpC-citrate complex possesses RNAP recruitment capability, we constructed a hybrid promoter (HP­(V2)) in which both CcpC binding sites were positioned upstream of the constitutive promoter *Plpp1.0* (Figure 2a). We hypothesized that the CcpC-citrate complex would enhance transcription by facilitating RNAP recruitment when positioned upstream of a constitutive promoter. Fluorescence assays revealed a clear citrate-dependent activation pattern. For all three CcpC homologues tested, GFP expression increased with increasing citrate concentrations (Figure 2b–d), reaching the highest fluorescence intensity at 80 mM citrate. Notably, the fluorescence levels were approximately 7.09-, 6.00-, and 3.99-fold higher than those of the PC, indicating strong transcriptional activation. The results demonstrated that CcpC-citrate complex functions as an RNAP recruiter and transcriptional enhancer.

**Figure 2:**
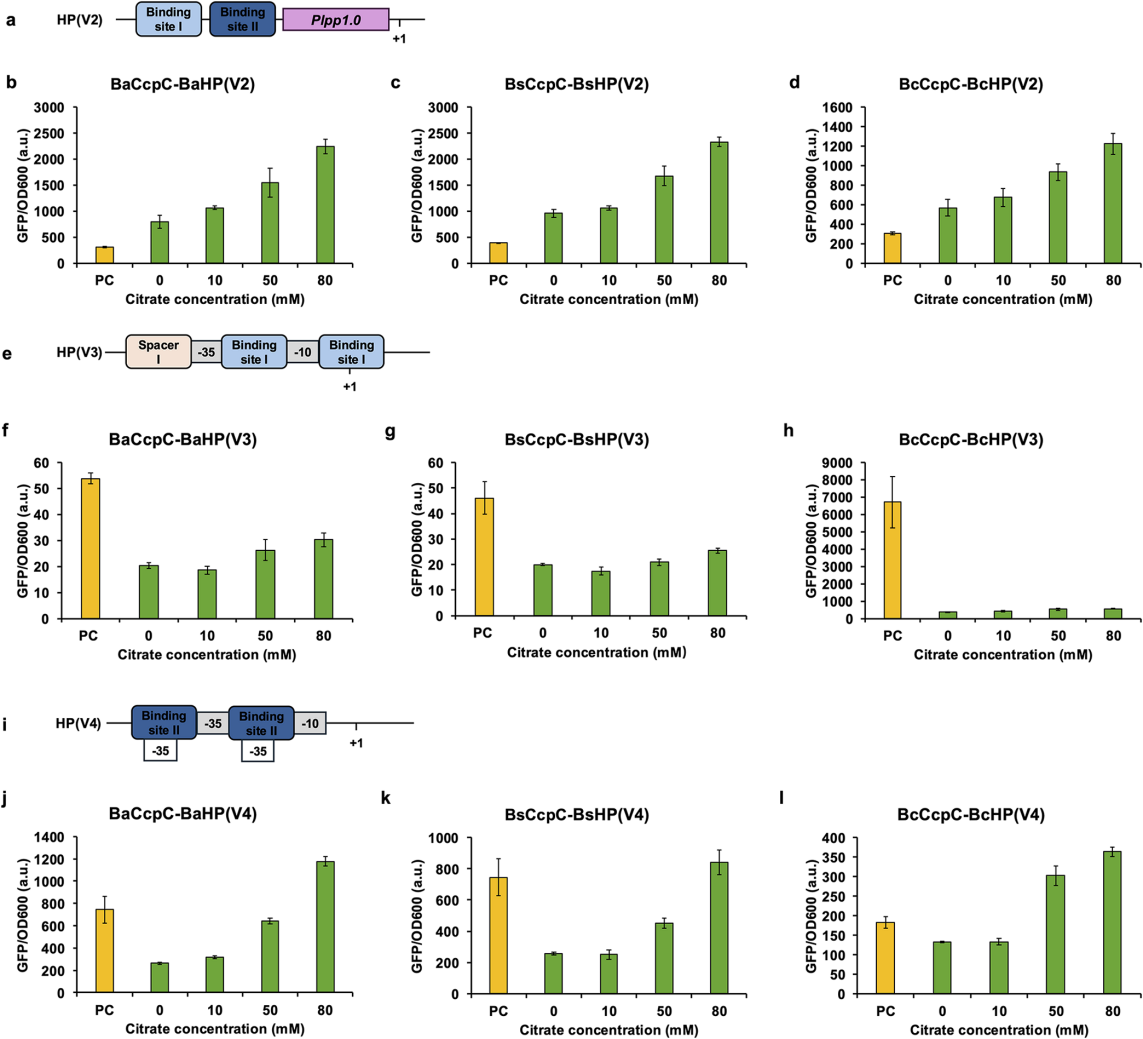
Functional characterization of CcpC binding sites using hybrid promoter designs. (a) Architecture of hybrid promoter V2 (HP­(V2)), in which both CcpC binding sites are positioned upstream of the *Plpp1.0* promoter to allow RNAP recruitment and transcription enhancement. (b–d) Fluorescence assays of the HP­(V2) under different citrate concentrations (0, 10, 50, and 80 mM). (e) Architecture of hybrid promoter V3 (HP­(V3)), in which two binding site I are positioned downstream of the −35 and −10 region (gray boxes) of *PL* promoter, enabling evaluation of the regulatory role of binding site I. (f–h) Fluorescence assays of the HP­(V3) under different citrate concentrations. (i) Architecture of hybrid promoter V4 (HP­(V4)), in which two binding sites II are positioned upstream of the −35 and −10 region of *PL* promoter to evaluate the regulatory contribution of binding site II. The white box indicates the −35 motif inside binding site II from *Bacillus*. (j–l) Fluorescence assays of the HP­(V4) under different citrate concentrations. PC indicates the positive control strain lacking CcpC-mediated repression. Data represent mean ± SD from three biological replicates (*n* = 3).

To examine the persistent binding of CcpC to binding site I, we constructed a second hybrid promoter (HP­(V3)) in which only binding site I was retained. Accordingly, two copies of binding site I were incorporated into a constitutive *PL* promoter and positioned downstream of the −35 and −10 regions, respectively (Figure 2e). We hypothesized that CcpC would remain bound to this engineered promoter and interfere with RNAP access, resulting in transcriptional repression. Fluorescence assays showed a strong repression pattern, with repression efficiencies of 65.45, 62.24, and 94.33% for three CcpC homologues, respectively (Figure 2f–h). Although GFP expression exhibited minor fluctuations across different citrate concentrations, the fluorescence levels remained significantly lower than those of the PC group, indicating sustained repression. These results indicated that binding site I functions as a stable anchoring element that maintains persistent CcpC association.

To investigate the dissociation of CcpC from binding site II upon citrate induction, we constructed another hybrid promoter (HP­(V4)) (Figure 2i). Two copies of binding site II were incorporated into the *PL* promoter and placed upstream of the −35 and −10 regions, respectively. We hypothesized that, in the absence of citrate, CcpC binding to this site interferes with RNAP access to the −35 region, resulting in transcriptional repression. Upon citrate induction, this interaction is weakened, thereby relieving repression. Fluorescence assays revealed citrate-responsive derepression patterns (Figure 2j–l). At low citrate concentrations, GFP expression remained relatively low, consistent with transcriptional repression. As citrate concentration increased, GFP expression gradually increased, indicating the relief of repression due to CcpC complex dissociation. Additionally, at high citrate concentration, the fluorescence intensity exceeded that of the PC group. This elevated expression might result from two synergistic effects. One is the presence of multiple −35 elements in the hybrid promoter, as the binding site II sequence itself contains a −35 motif that enhances RNAP binding. The other is that the CcpC-citrate complex actively recruits RNAP locally. Together, the results demonstrated that binding site II serves as a switch that modulates transcription through ligand-dependent dissociation of CcpC.

Overall, similar regulatory patterns were observed across the three CcpC homologues tested (Figure 2). Notably, BaCcpC and BsCcpC, which share higher sequence similarity, exhibited similar transcriptional responses across the engineered promoter variants, whereas BcCcpC displayed comparable trends with different signal intensities. These results suggested that the regulatory behavior of CcpC is largely conserved among these homologues.

### 2.4 Development of Citrate-Responsive CcpC-Based Biosensors in *E. coli*

Following the functional characterization of CcpC operators, we next sought to construct citrate-responsive biosensors. To achieve this goal, *PLBx* hybrid promoters (PLBa, PLBs, and PLBc) were designed by incorporating both binding site I and binding site II into the *PL* promoter scaffold (Figure 3a). In this design, the essential regulatory elements were extracted from the original promoter configuration (Figure 1d), while uncharacterized or redundant sequences were removed.

**Figure 3:**
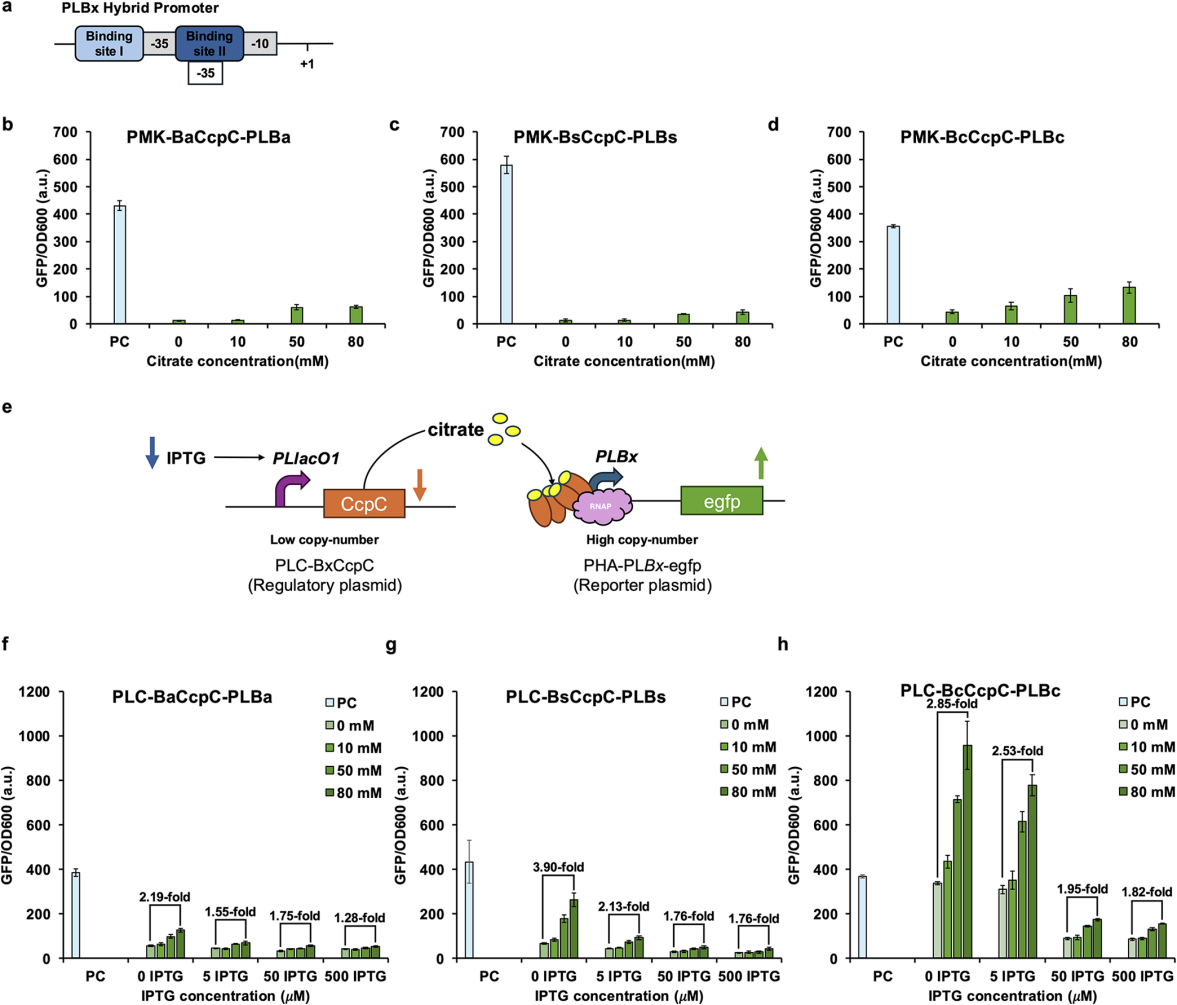
Design of *PLBx* hybrid promoters and tuning of citrate-responsive biosensors. (a) Design of the *PLBx* hybrid promoter. Two CcpC binding sites from the *PcitB* promoter are integrated into the *PL* promoter scaffold. (b–d) Fluorescence assays of *PLBx* promoters using CcpC homologues from three *Bacillus* species. Relative GFP fluorescence (GFP/OD600) was measured under different citrate concentrations (0, 10, 50, and 80 mM) under 0.5 mM IPTG induction. (e) Strategies for tuning CcpC expression levels by modulating plasmid copy number and IPTG induction, thereby reducing regulator abundance. (f–h) Fluorescence assays of *PLBx*-based biosensors under reduced CcpC expression conditions using low-copy-number plasmids or lower IPTG induction. Relative GFP fluorescence (GFP/OD600) was measured across different citrate concentrations (0, 10, 50, and 80 mM) and IPTG induction levels (0, 5, 50, and 500 μM). PC indicates the positive control lacking CcpC-mediated repression. Data represent mean ± SD from three biological replicates (*n* = 3).

Based on the regulatory mechanism of CcpC, we hypothesized that in the absence of citrate, CcpC binding would block the −35 region of the *PLBx* promoter, resulting in repression of GFP expression. Upon citrate induction, the CcpC-citrate complex would gradually dissociate from binding site II, thereby exposing the −35 region and facilitating RNAP access and recruitment, similar to the regulation mechanism observed in the native *PcitB* promoter. Fluorescence assays revealed the expected derepression upon citrate induction from all three CcpC homologues (Figure 3b–d). In the absence of citrate, strong repression of GFP expression was observed, whereas increasing citrate concentrations gradually relieved this repression. However, the overall fluorescence output and dynamic range of the biosensors remained relatively limited. We reasoned that this restricted dynamic range might result from excessive CcpC expression, which could strongly inhibit promoter activity by saturating the binding sites.

To improve biosensor performance, we next optimized tunable genetic parameters controlling CcpC expression. Specifically, we reduced intracellular CcpC abundance by lowering plasmid copy number and decreasing IPTG induction levels (Figure 3e). Reducing plasmid copy number alone did not significantly improve biosensor performance. However, further decreasing IPTG concentration substantially enhanced the dynamic range across all three CcpC homologues (Figure 3f–h). Notable improvements became apparent when the IPTG concentration was reduced to 5 μM. For the BaCcpC-PLBa system, dynamic ranges reached 1.55-fold under 5 μM IPTG induction and 2.19-fold without IPTG (Figure 3f). The BsCcpC-PLBs system exhibited dynamic ranges of 2.13-fold at 5 μM IPTG and 3.90-fold without IPTG (Figure 3g). The BcCcpC-PLBc system showed dynamic ranges of 2.53-fold at 5 μM IPTG and 2.85-fold without IPTG (Figure 3h). Consistent with previous observations, the fluorescence intensity exceeded that of the PC group at high citrate concentrations. This increase may be attributed to the synergistic effect of multi −35 elements and RNAP recruitment capability of CcpC-citrate complex.

Among the three systems, BcCcpC-PLBc provided the most favorable balance between dynamic range and output signal intensity (Figure 3h). Although slightly higher fluorescence output was observed in the IPTG absence group, we selected 5 μM IPTG for subsequent experiments. Because *PLlacO1* is an inducible promoter, relying solely on basal leakage may result in unstable CcpC expression. Maintaining a low but defined IPTG induction ensures more consistent regulator expression while preserving improved biosensor performance.

To further expand the biosensor repertoire, we constructed cross-species combinations of CcpC homologues and hybrid promoters (Figure 4a). This strategy enabled systematic assessment of biosensor performance across diverse regulator-promoter pairings. The results showed that BcCcpC consistently produced the highest fluorescence outputs and dynamic ranges when paired with different hybrid promoters, outperforming the other homologues (Figure 4b–d). Notably, the BcCcpC-PLBs biosensor exhibited the strongest fluorescence output among all tested pairs, with a dynamic range of 2.78-fold (Figure 4c). Together, these engineering efforts established a modular CcpC-based citrate biosensor platform and identified BcCcpC-PLBs as the most robust configuration for further characterization.

**Figure 4:**
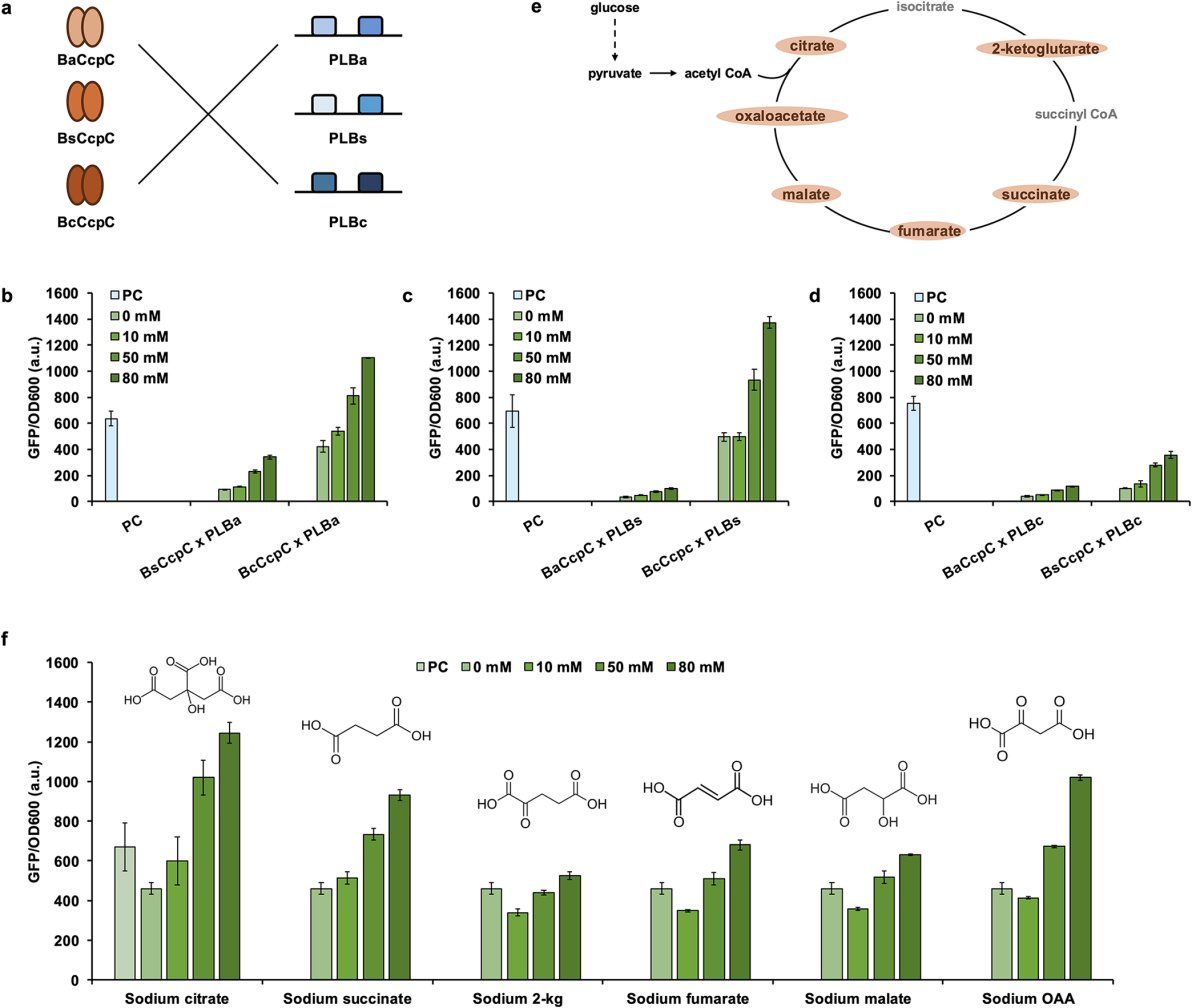
Cross-species regulator-operator pairing and ligand specificity test. (a) Design of cross-species regulator-operator pairings between CcpC homologues and *PLBx* promoters derived from different *Bacillus* species. (b–d) Fluorescence assays of biosensor pairings show improved biosensor performance. Relative GFP fluorescence (GFP/OD600) was measured across different citrate concentrations (0, 10, 50, and 80 mM) under 5 μM IPTG induction. (e) Schematic representation of the TCA cycle highlighting the metabolites evaluated in this study. (f) Ligand specificity of the BcCcpC-PLBs biosensor toward TCA cycle-related metabolites, including citrate, succinate, 2-ketoglutarate (2-kg), fumarate, malate, and oxaloacetate (OAA) in the form of sodium salt. PC indicates the positive control lacking CcpC-mediated repression. Data represent mean ± SD from three biological replicates (*n* = 3).

### 2.5 Ligand Scope of BcCcpC-PLBs Biosensor System

To investigate the ligand specificity of the engineered biosensor, we next examined the response of the BcCcpC-PLBs system to several metabolites from the TCA cycle. Six representative intermediates, including citrate, succinate, 2-ketoglutarate (2-kg), fumarate, malate, and oxaloacetate (OAA) (Figure 4e), were tested and fed in the form of sodium salt to evaluate the ligand scope of the biosensor.

Fluorescence assays showed that the biosensor responded to all tested metabolites to varying degrees (Figure 4f). Among them, citrate exhibited the strongest dynamic range of 2.70-fold. OAA produced the second highest dynamic range of 2.22-fold, followed by succinate with a 2.02-fold dynamic range. Additionally, 2-ketoglutarate, fumarate, and malate induced relatively weaker responses. The responsiveness of the biosensor to multiple TCA intermediates suggested that BcCcpC exhibited ligand promiscuity toward structurally related metabolites. Many TCA intermediates share similar carboxylate-rich structures, which may allow partially interaction with the ligand-binding pocket of BcCcpC. Despite this broader responsiveness, the biosensor displayed the highest sensitivity to citrate, indicating that citrate remains the dominant ligand for BcCcpC.

### 2.6 Engineering of BcCcpC Regulator for TCA Cycle Metabolites Sensing

To further improve the dynamic performance of the biosensor, we next engineered the BcCcpC regulator to enhance its responsiveness to TCA cycle metabolites. We first predicted the structure of BcCcpC using AlphaFold3 and aligned the predicted model with the crystal structure of BaCcpC (PDB: 7DMW) (Figure 5a). Structural alignment revealed highly conserved ligand-binding pockets between the two homologues. To identify key residues involved in ligand recognition, citrate was docked into the predicted BcCcpC structure. Docking analysis suggested that several residues, including S138, R156, S198, S200, and R264, are located near the citrate molecule and likely participate in ligand-protein interactions (Figure 5b). Based on these structural insights, we performed site-directed mutagenesis at these five residues to explore variants with improved sensing performance. Screening of the resulting mutant library identified 10 variants that showed improved responses to citrate compared with the wild type (WT) (Figure 5c). Among these, two variants, S138L and S138R, displayed the most significant improvements in fluorescence intensity and dynamic range.

**Figure 5:**
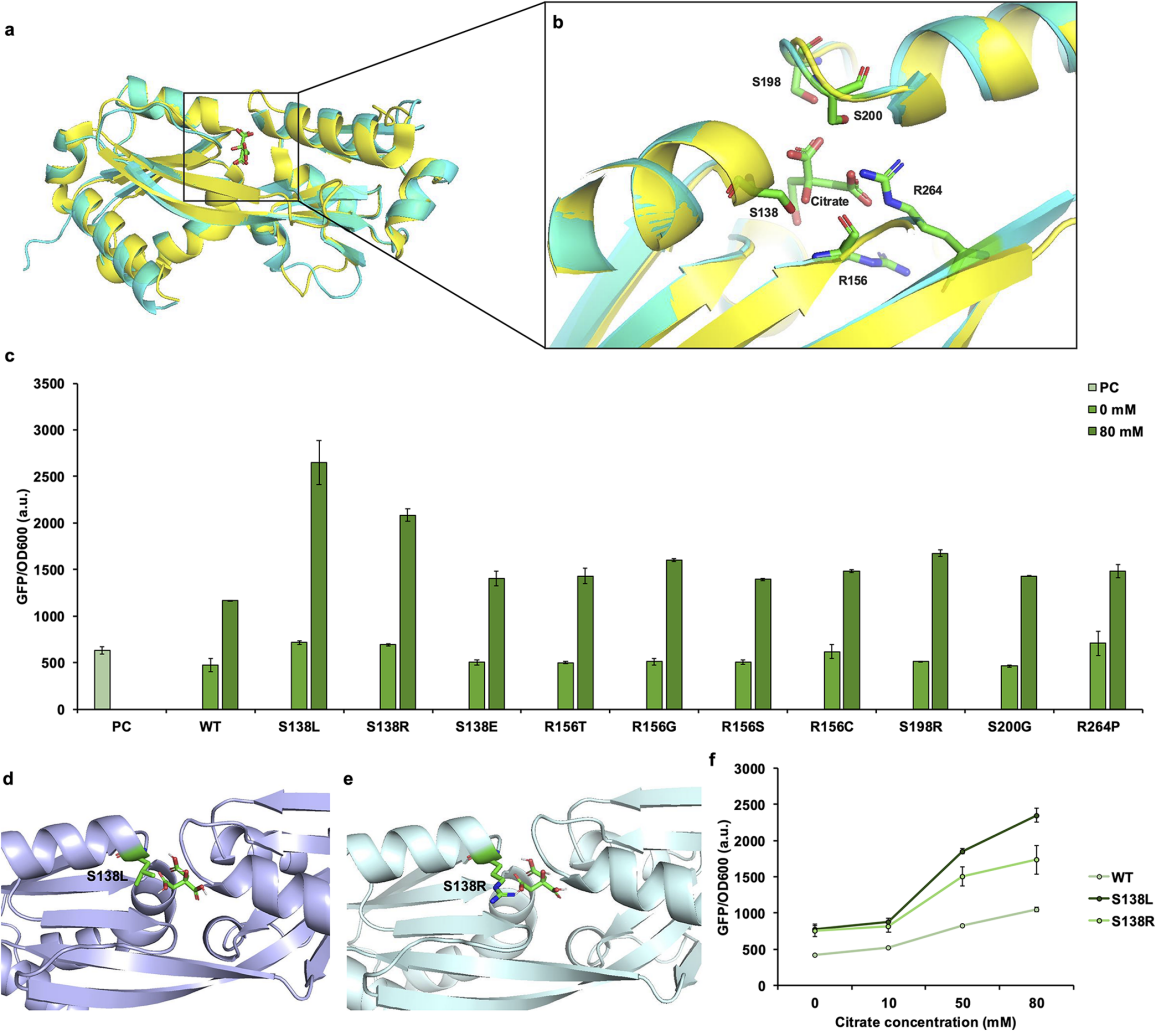
Structure-guided engineering of BcCcpC enhances the dynamic range of citrate-responsive biosensor. (a) Structural alignment of BcCcpC (cyan) with BaCcpC (yellow; PDB: 7DMW). (b) Predicted citrate-binding pocket of BcCcpC highlighting key interacting residues (S138, R156, S198, S200, and R264). (c) Screening of BcCcpC variants generated by targeted mutagenesis at the predicted ligand-binding residues. (d, e) Structural modeling of top-performing variants S138L (d) and S138R (e) in complex with citrate. (f) Dynamic ranges of WT, S138L, and S138R toward citrate. Relative GFP fluorescence (GFP/OD600) was measured across different citrate concentrations (0, 10, 50, and 80 mM) under 5 μM IPTG induction. PC indicates the positive control lacking CcpC-mediated repression. Data represent mean ± SD from three biological replicates (*n* = 3).

To understand the molecular basis underlying the enhanced performance of these variants, we performed computational simulations on the S138L and S138R mutants (Figure 5d, e). Molecular docking analysis revealed that both mutations resulted in lower Vina scores (Table S2), indicating stronger predicted binding affinity between BcCcpC and citrate. In addition, cavity analysis showed a reduction in ligand-binding pocket volume in the mutant structures compared with the wild type (Table S2). These structural changes likely improve ligand accommodation and stabilize the ligand-protein interaction, thereby enhancing citrate responsiveness. Next, a detailed characterization of selected BcCcpC mutants was performed using gradient citrate concentrations as the inducer. The BcCcpC­(S138L)-PLBs biosensor showed the highest fluorescence output and greatest dynamic range, reaching 3.02-fold activation at 80 mM citrate (Figure 5f). Additionally, we further evaluated the responses of these variants to other TCA cycle metabolites. Compared with the wild-type BcCcpC, the engineered variants (S138L and S138R) exhibited slightly reduced responses to sodium 2-kg and fumarate and comparable responses to sodium OAA, succinate, and malate (Figure S1). The results indicated that the engineered variants maintain a strong sensing preference toward citrate.

## 3 Conclusions

Metabolite-responsive TF-based biosensors are powerful regulatory toolkits in MCFs. In this study, we engineered citrate-responsive CcpC-based biosensor systems by mining CcpC homologues from three *Bacillus* species. By constructing a series of hybrid promoters, we dissected the functional roles of the two binding sites within the CcpC-regulated *PcitB* promoter. Our results suggested that binding site I primarily functions as a stable anchoring site for the CcpC complex, whereas binding site II acts as a citrate-responsive switch. Through this hybrid promoter engineering strategy, we gained deeper insights into the functions of the CcpC operator. This improved our understanding of operator-regulator interactions and facilitated the development of citrate-responsive biosensors.

Building upon this mechanistic understanding, we developed citrate-responsive biosensors in *E. coli*. By mimicking the native regulatory configuration of the *PcitB* promoter while removing redundant sequences, we created *PLBx* hybrid promoters capable of translating citrate concentration into transcriptional output. Optimization of CcpC abundance further improved biosensor performance. Additionally, systematic pairing of different CcpC homologues and hybrid promoters indicated that BcCcpC-PLBs exhibited superior regulatory performance. Additionally, the engineered biosensor responded to multiple TCA cycle intermediates, but with astrong ligand preference toward citrate. This ligand promiscuity suggested that the current CcpC-based biosensor not only responds to citrate but also reflects broader metabolic states within the TCA cycle, which could be advantageous for monitoring overall TCA cycle activity but compromise specific metabolite-based regulation. Furthermore, structure-guided protein engineering of the BcCcpC regulator further improved biosensor responsiveness toward citrate. Mutation BcCcpC­(S138L) significantly improved the dynamic range to 3.02-fold. Computational simulations further suggested that these mutations strengthen ligand binding by altering the geometry of the ligand-binding pocket, as indicated by smaller cavity volumes.

In conclusion, this work expanded the current repertoire of metabolite-responsive biosensors and provides a new approach for sensing central carbon metabolites. Future efforts will focus on improving ligand selectivity and enhancing dynamic range through structure-guided protein engineering and promoter engineering. Moreover, this study provides a foundation for developing citrate-responsive regulatory systems for fine-tuning microbial cell factories.

## 4 Materials and Methods

### 4.1 Strains, Plasmids, and Chemicals

All strains and plasmids used in this study are listed in Supporting Information Table S1. The *E. coli* strain XL1-blue (Stratagene, La Jolla, CA) was used for plasmid construction and extraction, while *E. coli* BW25113 (CGSC) was used for *in vivo* CcpC biosensor characterization. Plasmids pHA-MCS (high-copy number), pMK-MCS (medium-copy number), and pLC-MCS (low-copy number) were used as plasmid vectors for gene expression. CcpC homologues and their corresponding promoters were obtained from the genomic DNA of *B. amyloliquefaciens* *ATCC 23350*, *B. subtilis* *168*, and *B. cereus* *ATCC 14579*. All chemicals were procured from Sigma-Aldrich. Phusion DNA polymerase, restriction endonucleases, and the Quick Ligation Kit were purchased from New England Biolabs (Beverly, MA, USA). The Plasmid Miniprep Kit, Gel Recovery Kit, and DNA Cleanup Kit were obtained from Zymo Research (Irvine, CA, USA).

### 4.2 Plasmid Construction

CcpC homologues were inserted into the plasmids pMK-MCS or pLC-MCS between *Kpn*I/*Bam*HI restriction sites, generating pMK-pLlacO1-BaCcpC, pMK-pLlacO1-BsCcpC, pMK-pLlacO1-BcCcpC, pLC-pLlacO1-BaCcpC, pLC-pLlacO1-BsCcpC, and pLC-pLlacO1-BcCcpC, respectively.

Reporter plasmids were constructed by inserting *PcitB* promoters amplified from the genomes of *B. amyloliquefaciens* *ATCC 23350*, *B. subtilis* *168*, and *B. cereus* *ATCC 14579*. into the high-copy pHA-eGFP-MCS plasmid using XhoI/*Eco*RI restriction sites to replace the *PLlacO1* promoter, resulting in pHA-BaPcitB-eGFP, pHA-BsPcitB-eGFP, and pHA-BcPcitB-eGFP, respectively. Hybrid promoters (V2, V3, V4, and *PLBx*) were obtained by PCR amplification of designed sequences, followed by digestion with XhoI/*Eco*RI, and subsequent cloning into pHA-eGFP-MCS.

### 4.3 Stain Cultivation

*E. coli* strains were cultivated at 37 °C in Luria–Bertani (LB) medium containing 5 g/L yeast extract, 10 g/L NaCl, and 10 g/L tryptone, supplemented with the appropriate antibiotics when necessary. For fluorescence assays, 3 mL of M9Y medium (containing 6 g/L Na_2_HPO_4_, 0.5 g/L NaCl, 3 g/L KH_2_PO_4_, 1 g/L NH_4_Cl, 1 mM MgSO_4_, 0.1 mM CaCl_2_, 20 g/L glucose and 5 g/L yeast extract) was inoculated with 1% (v/v) seed culture and cultivated in a rotary shaker at 270 rpm for 24 h. When required, ampicillin (Amp), kanamycin (Kan), and chloramphenicol (Cl) antibiotics were added to the medium at final concentrations of 100, 50, and 34 μg/mL, respectively. Isopropyl β-D-1-thiogalactopyranoside (IPTG) and sodium citrate, or other inducers, were added at the concentrations specified above.

### 4.4 Characterization of CcpC-Based Biosensor Performance

Transformants were plated on LB agar plates supplemented with appropriate antibiotics and incubated at 37 °C overnight. Single colonies were selected and inoculated into 3 mL LB medium containing appropriate antibiotics and cultured overnight as seed cultures. 1% (v/v) seed culture, IPTG, and the corresponding inducers were added to 3 mL M9Y medium. After 24 h cultivation, 20 μL of culture was mixed with 180 μL distilled water in a black 96-well plate for measurement of cell density and fluorescence using a Synergy microplate reader (BioTek, Winooski, VT). Cell density was measured at 600 nm (OD600) wavelength. Green fluorescence (GFP) was measured with an excitation wavelength of 485 nm and an emission wavelength of 528 nm. The GFP/OD600 ratio was calculated as previously described and was measured in arbitrary units (a.u.).^8,31^ All experiments were performed with three biological replicates (*n* = 3).

### 4.5 Site-Directed Mutagenesis of BcCcpC and Molecular Docking

The BcCcpC protein structure was predicted by AlphaFold 3.^32^ The molecular docking was performed by cavity-detection guided blind docking, CB-Dock2.^33^ The visualization of the structures was in PyMOL.^35^ Site-directed mutation of BcCcpC was carried out by using plasmid pLC-PLlacO1-BcCcpC as the template. The BcCcpC variants were constructed using the SLIM method^34^ and verified by Sanger sequencing.

## Supplementary Material



## Notes

Additional experimental methods and results, including strains and plasmids used in this study, docking analysis data for BcCcpC and its variants, and dynamic ranges of BcCcpC and its variants toward representative TCA cycle metabolites (PDF)
