# Supplementary material for: Engineering the Bacillus Transcription Factor CcpC to Construct Citrate-Responsive Biosensors in Escherichia coli
Source: ACS Synth Biol. 2026 May 26;15(6):2649–59. doi: 10.1021/acssynbio.6c00278 (PMC13288900; doi:10.1021/acssynbio.6c00278)
Supplement: Supplementary file 1 [file sb6c00278_si_001.pdf]

Supplementary Information

Engineering the *Bacillus* Transcription Factor CcpC to Construct Citrate-Responsive Biosensors in *Escherichia coli*

Xinyu Gong[1], Shuo Yu[1], Jiyang Zhang[1], Zebang Chen[1], Jianli Zhang[1], Qi Gan[1], Yajun Yan[1,*]

1. School of Chemical, Materials and Biomedical Engineering, College of Engineering, The University of Georgia, Athens, GA 30602, USA.

*Correspondence: yajunyan@uga.edu

Table S1. Strains and Plasmids used in this study.

Strains	Description	Reference
E. coli XL-1 Blue	*recA1 endA1 gyrA96 thi-1 hsdR17 supE44 relA1 lac F' [traD36 proAB lacIqZΔM15 Tn10 (Tetr)]*	Stratagene
E. coli BW25113	*rrnBT14 Δlac ZWJ16 hsdR514 ΔaraBADAH33 ΔrhaBADLD78*	CGSC
**Plasmids**	**Description**	**Reference**
pHA-MCS	*PLlacO1*, multiple cloning sites, *ColE1 ori, Amp*^{R}	[1]
pHA-eGFP-MCS	*PLlacO1*, eGFP, multiple cloning sites, *ColE1 ori, Amp*^{R}	[2]
pMK-MCS	*PLlacO1*, multiple cloning sites, *p15Aori, Kan*^{R}	[3]
pLC-MCS	*PLlacO1*, multiple cloning sites, *pSC101* ori, Cl*^{R}	This study
pHA-plpp1.0-eGFP-MCS	*PLpp1.0*, eGFP, multiple cloning sites, *ColE1 ori, Amp*^{R}	This study
pHA-BaPcitB(V1)-eGFP	pHA harboring wild type PcitB from *Bacillus amyloliquefaciens ATCC 23350* driving egfp expression	This study
pHA-BsPcitB(V1)-eGFP	pHA harboring wild type PcitB from *Bacillus subtilis 168* driving egfp expression	This study
pHA-BcPcitB(V1)-eGFP	pHA harboring wild type PcitB from *Bacillus cereus ATCC14579* driving egfp expression	This study
pHA-BaHP(V2)-eGFP	pHA harboring BaCcpC binding sites I&II before PLpp1.0 promoter driving egfp expression	This study
pHA-BsHP(V2)-eGFP	pHA harboring BsCcpC binding sites I&II before PLpp1.0 promoter driving egfp expression	This study
pHA-BcHP(V2)-eGFP	pHA harboring BcCcpC binding sites I&II before PLpp1.0 promoter driving egfp expression	This study
pHA-BaHP(V3)-eGFP	pHA harboring hybrid promoter of BaCcpC binding site I and PL driving egfp expression	This study
pHA-BsHP(V3)-eGFP	pHA harboring hybrid promoter of BsCcpC binding site I and PL driving egfp expression	This study
pHA-BcHP(V3)-eGFP	pHA harboring hybrid promoter of BcCcpC binding site I and PL driving egfp expression	This study
pHA-BaHP(V4)-eGFP	pHA harboring hybrid promoter of BaCcpC binding site II and PL driving egfp expression	This study
pHA-BsHP(V4)-eGFP	pHA harboring hybrid promoter of BsCcpC binding site II and PL driving egfp expression	This study
pHA-BcHP(V4)-eGFP	pHA harboring hybrid promoter of BcCcpC binding site II and PL driving egfp expression	This study
pHA-PLBa-eGFP	pHA harboring hybrid promoter of BaCcpC binding site I&II and PL driving egfp expression	This study
pHA-PLBs-eGFP	pHA harboring hybrid promoter of BsCcpC binding site I&II and PL driving egfp expression	This study
pHA-PLBc-eGFP	pHA harboring hybrid promoter of BcCcpC binding site I&II and PL driving egfp expression	This study
pMK-PLlacO1-BaCcpC	pMK harboring wild type CcpC from *Bacillus amyloliquefaciens ATCC 23350* driving egfp expression	This study

pMK-PLlacO1-BsCcpC	pMK harboring wild type CcpC from *Bacillus subtilis 168* driving egfp expression	This study
pMK-PLlacO1-BcCcpC	pMK harboring wild type CcpC from *Bacillus cereus ATCC14579* driving egfp expression	This study
pLC-PLlacO1-BaCcpC	pLC harboring wild type CcpC from *Bacillus amyloliquefaciens ATCC 23350* driving egfp expression	This study
pLC-PLlacO1-BsCcpC	pLC harboring wild type CcpC from *Bacillus subtilis 168* driving egfp expression	This study
pLC-PLlacO1-BcCcpC	pLC harboring wild type CcpC from *Bacillus cereus ATCC14579* driving egfp expression	This study
pLC-PLlacO1-BcCcpC (S138L)	pLC-PLlacO1-BcCcpC with S138L mutation at BcCcpC	This study
pLC-PLlacO1-BcCcpC (S138R)	pLC-PLlacO1-BcCcpC with S138R mutation at BcCcpC	This study
pLC-PLlacO1-BcCcpC (S138E)	pLC-PLlacO1-BcCcpC with S138E mutation at BcCcpC	This study
pLC-PLlacO1-BcCcpC (R156T)	pLC-PLlacO1-BcCcpC with R156T mutation at BcCcpC	This study
pLC-PLlacO1-BcCcpC (R156G)	pLC-PLlacO1-BcCcpC with R156G mutation at BcCcpC	This study
pLC-PLlacO1-BcCcpC (R156S)	pLC-PLlacO1-BcCcpC with R156S mutation at BcCcpC	This study
pLC-PLlacO1-BcCcpC (R156C)	pLC-PLlacO1-BcCcpC with R156C mutation at BcCcpC	This study
pLC-PLlacO1-BcCcpC (S198R)	pLC-PLlacO1-BcCcpC with S198R mutation at BcCcpC	This study
pLC-PLlacO1-BcCcpC (S200G)	pLC-PLlacO1-BcCcpC with S200G mutation at BcCcpC	This study
pLC-PLlacO1-BcCcpC (R264P)	pLC-PLlacO1-BcCcpC with R264P mutation at BcCcpC	This study

Table S2. Docking analysis of BcCcpC and its variants.

**Protein**	**Ligand**	**Vina score**	**Cavity volume (Å^3)**
BcCcpC_WT	Citrate	-6.2	509
BcCcpC_S138R		-6.4	223
BcCcpC_S138L		-7.5	303

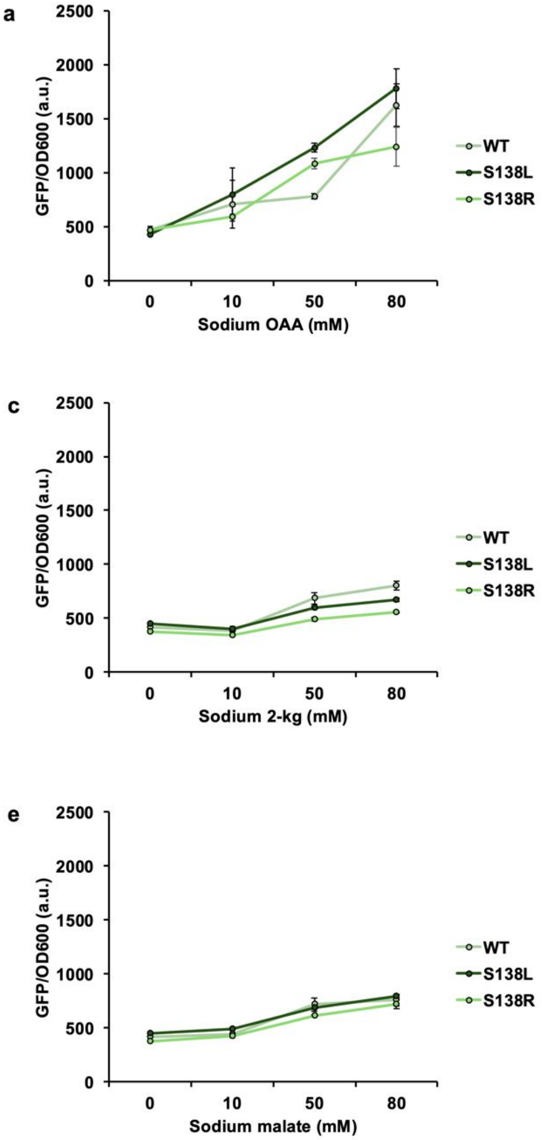

Figure S1. Dynamic range of WT, S138L, S138R towards 0, 10, 50, or 80 mM (a) sodium OAA, (b) sodium succinate, (c) sodium 2-kg, (d) sodium fumarate, and (e) sodium malate. All data are reported as mean ± SD from three biological replicates (n = 3).

Reference:

(1) Jiang, T.; Teng, Y.; Li, C.; Gan, Q.; Zhang, J.; Zou, Y.; Desai, B. K.; Yan, Y. Establishing Tunable Genetic Logic Gates with Versatile Dynamic Performance by Varying Regulatory Parameters. *ACS Synth. Biol.* **2023**, *12* (12), 3730–3742. https://doi.org/10.1021/acssynbio.3c00554.

(2) Li, C.; Zhou, Y.; Zou, Y.; Jiang, T.; Gong, X.; Yan, Y. Identifying, Characterizing, and Engineering a Phenolic Acid-Responsive Transcriptional Factor from *Bacillus Amyloliquefaciens*. *ACS Synth. Biol.* **2023**, *12* (8), 2382–2392. https://doi.org/10.1021/acssynbio.3c00206.

(3) Teng, Y.; Gong, X.; Zhang, J.; Obideen, Z.; Yan, Y. Investigating and Engineering an 1,2-Propanediol-Responsive Transcription Factor-Based Biosensor. *ACS Synth. Biol.* **2024**, *13* (7), 2177–2187. https://doi.org/10.1021/acssynbio.4c00237.
